# An analysis of e-cigarette policy action, inaction and industry influence: implications for youth uptake in New Zealand

**DOI:** 10.1177/17579139251322009

**Published:** 2025-03-18

**Authors:** L Hardie, B Freeman, J McCool

**Affiliations:** School of Population Health, The University of Auckland, Private Bag 92019, Auckland 1142, New Zealand; School of Public Health, The University of Sydney, Sydney, NSW, Australia; School of Population Health, The University of Auckland, Auckland, New Zealand

**Keywords:** tobacco, e-cigarettes, youth

## Abstract

**Aims:**

In 2024, New Zealand had one of the highest rates of e-cigarette use among youth globally. In this article, we aim to examine key developments in e-cigarette policy that may have contributed to high uptake among young people in New Zealand between 2015 and 2024. By identifying key policy-relevant documents by government agencies and interactions with the industry, we aim to offer insights for jurisdictions looking to implement or strengthen e-cigarette policies.

**Methods:**

We searched publicly available New Zealand government websites to identify documents related to e-cigarette policy between 2015 and 2024 (Ministry of Health, New Zealand Parliament, Beehive, Courts of New Zealand and New Zealand Customs). We included key policy-related documents for analysis. Documents were organised and summarised sequentially into a timeline graphic and chronological narrative results.

**Results:**

New Zealand introduced policies aimed at reducing youth e-cigarette uptake slowly compared to other high-income countries such as the UK and Australia. When policies were introduced, they lacked strength, which enabled the e-cigarette and tobacco industry to oppose, bypass and, ultimately, weaken the impact of such policies. The e-cigarette industry had multiple interactions with public health actors that may have positioned the industry as a legitimate partner in tobacco harm reduction.

**Conclusion:**

This study highlights that jurisdictions must move quickly to introduce effective measures on e-cigarettes to protect health. Policies must be sufficiently comprehensive to prevent the industry from opposing and bypassing laws. Governments must protect policy processes from companies that profit from nicotine addiction in line with the WHO Framework on Tobacco Control.

## Introduction

E-cigarette use (vaping) has escalated globally since its popularisation around 2015. Of particular concern is the rise in use among young people who may never have smoked.^1^ However, developing effective legislation is complicated as decision-makers also consider the potential benefits to people who smoke and use e-cigarettes to quit. Traditionally, nicotine replacement therapies, such as nicotine gum, are governed by therapeutic standards and protocols. E-cigarettes, however, are commonly marketed as consumer products, meaning mechanisms and safeguards to control the use and distribution of therapeutic goods are not generally applied to e-cigarettes in many countries, such as the UK^2^ and the US.^3^

Approaches to e-cigarette policymaking vary^4^ between jurisdictions. Some nations, such as Palau and Cambodia, have implemented complete bans on these products.^5,6^ Australia has taken a more medicalised approach and legislated e-cigarettes as pharmacy-only medicines in 2024,^7^ and other countries have varied combinations. For example, liquid-based e-cigarettes are prohibited in Japan, but heated tobacco vaping products are permitted.^8^ The European Union issued a directive on e-cigarette regulation in 2014, which included, for example, marketing, sponsorship and nicotine concentrations.^9^ One possible explanation for the diverse policies across jurisdictions is the lack of long-term population-level evidence on e-cigarette harms and benefits.

The New Zealand government first introduced e-cigarette legislation in 2020. By 2024, rates of e-cigarette use among young people in New Zealand were among the highest globally.^10^ In 2024, the New Zealand Health Survey reported daily vaping among 15- to 17-year-olds at 10.5%.^11^ Among 18- to 24-year-olds, daily use was 26.5% in 2024, with declines in smoking in both groups far outstripped by uptake in e-cigarette use.^12^ Of particular concern is the high rates of use among young Ma-ori (indigenous New Zealanders). According to one survey, 22.3% of Ma-ori year 10 students (aged 14–15 years) vape daily, more than double the rate among the general cohort of year 10 students.^13^

This article describes the development of e-cigarette policy in New Zealand, in particular, key policy-relevant events and the role of the e-cigarette industry in attempting to influence these policies. This case may provide countries that have yet to develop e-cigarette policies or seek to strengthen them with guidance on critical influences and actions that may impact e-cigarette use at a population level.

## Methods

To explore policy changes over time, we searched publicly available documents related to e-cigarette policy from New Zealand Government websites. This approach was chosen to provide a detailed, chronological description of e-cigarette policy developments. We scanned government websites to identify policy documents related to e-cigarettes between 2015 and November 2024 to capture the relevant period from when e-cigarettes were first available in New Zealand. Websites included the New Zealand Ministry of Health,^14^ New Zealand Parliament,^15^ Beehive (New Zealand Government official website),^16^ Courts of New Zealand^17^ and New Zealand Customs Service.^18^ We included policy-relevant documents, press releases, official reports, regulations, court decisions and legislation documents. Information relevant to clinical practices, technical product notification and manufacturing information was excluded. We also collected industry interactions with the government or health sector, including letters, legal cases and group membership. The industry is defined as vape or tobacco companies.

**Figure 1:**
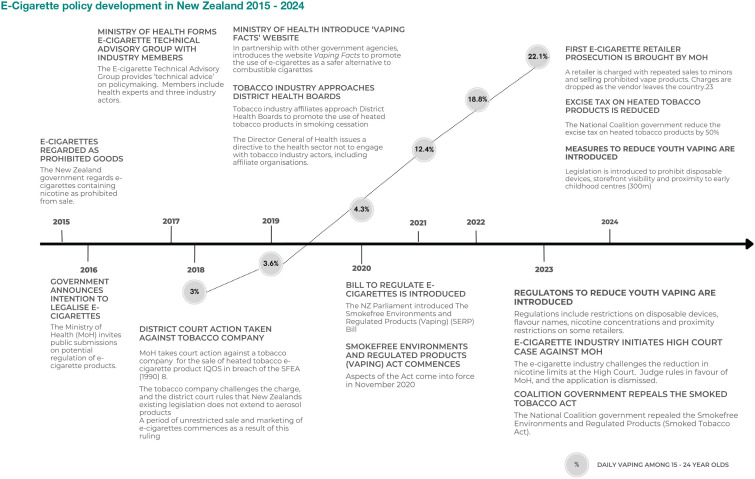
E-Cigarette policy development in New Zealand 2015 - 2024

Using key search terms like ‘e-cigarette’, ‘policy’ and derivatives (Table 1), we identified relevant documents. The documents and data were extracted from the websites using content analysis methods. Key details, such as dates, titles, content and authorship, were recorded. The data are summarised in Figure 1, organised in chronological order. The last search was conducted in November 2024. Using data from the New Zealand Health Survey, we also plotted the prevalence of daily e-cigarette use (vaping) among 15- to 24-year-olds during this period.^12^ A detailed summary of the findings is available in Supplemental Table 1. Narrative results are summarised chronologically.

**Table 1:** Search terms for policy documents related to e-cigarettes.

E-cigarette	Policy
Vape	Regulation
Vaping	Legislation
Electronic nicotine delivery systems	Report
ENDS	Bill
Heated tobacco products	Act
HTP	

## Results

We identified 26 documents relating to e-cigarette policy developments between 2015 and 2024 (Table 2). Key policy documents included two legislative bills (2020, 2024), two legislative acts (1990, 2020) and a set of regulations (2023). Two legal cases involving e-cigarette or tobacco companies were also identified (2018, 2023). Other documents consisted of reports (*n* = 6), press releases (*n* = 4), letters (*n* = 3), consultation documents (*n* = 2), a website, a supplementary order paper, a customs notice and a terms of reference document. The findings are summarised as a sequential narrative, and a visual timeline represents significant policy stages (Figure 1); further details are presented in Supplemental Table 1.

**Table 2:** E-cigarette policy-related documents included for analysis.

Document type	*N*
Report	6
Press release	4
Letter	3
Legislation (bills)	2
Legislation (acts)	2
Legal case (court)	2
Consultation documents	2
Terms of reference	1
Website	1
Supplementary order paper	1
Regulations	1
Notice	1
Total	26

### 2015: E-cigarettes as prohibited goods

In 2015, the New Zealand Government regarded e-cigarettes as prohibited goods. The Smoke-Free Environments Act (SFEA; 1990) and the Medicines Act (1981) were considered the key legislation which covered e-cigarettes.^19,20^ As the New Zealand government developed these policies before the emergence of e-cigarettes, the sale and supply of these products were covered by indirect clauses. Specifically, under the Act (1990), e-cigarettes containing nicotine were treated as tobacco products (as nicotine is traditionally derived from the tobacco leaf, although synthetic production without the tobacco leaf is common). The Act (1990) stipulated that ‘oral tobacco products’ were prohibited, a point that the industry later contested.^21^ Under the Medicines Act (1981), nicotine was classified as a scheduled medicine.^22^ Importantly, therapeutic claims such as that e-cigarettes can be used for smoking cessation were considered a breach of the Medicines Act without prior approval for this purpose.

### 2016: The Ministry of Health’s ‘precautionary’ approach to e-cigarettes

The Ministry of Health (MoH) described its approach to e-cigarettes in 2016 as ‘precautionary’, accounting for the limited evidence for e-cigarette efficacy in smoking cessation and potential health impacts.^22^ One MoH document stated that ‘the sale and supply of nicotine containing e-cigarettes are prohibited’.^22^ However, the same document outlined that importation was permitted for personal use as a smoking cessation tool, that the Act (1990) did not prohibit vaping in legislated smoke-free areas and that use was at the discretion of individual organisations. These ambiguities meant that the MoH conceded that laws were ‘confusing’ and ‘not routinely enforced’.^22^

### 2017: government signals its intention to introduce e-cigarette-specific legislation, consults industry

The New Zealand National Government announced its intention to revise the national tobacco control legislation (The SFEA, 1990) to include e-cigarette products in 2017.^23^

The MoH formed an e-cigarette ‘technical advisory group’ to provide expertise for policy and product design, ingredients, safety, packaging and labelling.^24^ Three members (of nine) were e-cigarette industry representatives^25^ who contravened recommendations made by the sixth WHO Conference of the Parties to protect e-cigarette policy from industry influence.^26^

### 2018: MoH’s unsuccessful court case against tobacco company

In 2018, the MoH brought charges against a tobacco company in New Zealand for the sale of heated tobacco e-cigarette products in the previous year,^21^ which was considered a breach of the existing legislation. The clause stipulated that ‘No person shall import for sale, sell, pack or distribute any tobacco product labelled or otherwise described as suitable for chewing, or for any other oral use (other than smoking)’.^19^ The Ministry argued that the text ‘other than smoking’ covered heated tobacco e-cigarette products. The tobacco company’s lawyers countered that the intention of the Act (1990) was never to capture aerosol products but for tobacco products chewed or otherwise consumed orally. The judge ruled in their favour that aerosol (vape) products were not legally captured by section 29(2), and the case was dismissed.^21^ The court ruling led to a liberalised retail environment where e-cigarettes were largely unrestricted; for example, there was no legislation to mandate a minimum purchase age or restrict marketing strategies, and retailers took advantage of this regulatory void with aggressive marketing.^27,28^

### 2019: MoH promotes e-cigarettes for smoking cessation and tobacco industry approaches

In 2019, in collaboration with the Health Promotion Agency, the MoH introduced a website entitled ‘Vaping Facts’^29^ and a $1.6 million ‘Quit Strong’ advertising campaign, which served to inform the New Zealand public that e-cigarettes were safer than combustible cigarettes and part of the suite of tools available to help smokers quit.^30^ Although this action was not specific to policy, it contrasted with the previous actions of the MoH, which brought charges against a tobacco company for the sale of heated tobacco vape products^21^ 1 year prior. To date, no company has attempted to gain Medsafe approval for e-cigarettes for smoking cessation purposes. Therefore, therapeutic claims such as that e-cigarettes can help smokers quit appeared to be in breach of the Medicines Act.

In the same year, a group^31^ funded solely by a tobacco company through a foundation^32^ approached several health boards and organisations to promote heated tobacco e-cigarette products. The then Director General of Health sent a letter to the health sector reminding them of the obligations under the World Health Organization Framework Convention on Tobacco Control (WHO FCTC). Specifically, Article 5.3, which cautions against interactions with commercial and other vested interests of the tobacco industry.^33^

### 2020: New Zealand government proposes legislation

In March 2020, the Labour Government proposed e-cigarette-specific legislation with the policy aimed ‘to support smokers to switch to these less harmful products’ and ‘balanced’ with measures to protect youth from uptake.^34^ The Smokefree Environments and Regulated Products (Vaping) Bill^35^ outlined key areas for restrictions on e-cigarette products, which included (1) a minimum purchase age of 18 years, (2) an extension of existing smoke-free areas to include vaping, (3) restrictions on advertising and sponsorship, (4) a restriction of flavours for sale in general retail settings (such as convenience stores and gas stations) to mint, menthol and tobacco and (5) a registration scheme for specialist retailers (who would be permitted greater advertising freedoms and to stock a full range of flavours).

The Health Committee received over a thousand submissions on the bill, including from the tobacco industry, e-cigarette manufacturers, community groups and health organisations.^36^ Following the consultation, supplementary order papers were put forward to introduce amendments to the bill. These included a delay to the enactment of the legislation and the relaxation of requirements for specialist retailer registration. No health organisations sought these amendments.^37^ The bill passed into law in August 2020^38^ and was enacted over a period of months.

### 2022: e-cigarettes for ‘recreational’ use and industry collaboration

In 2022, the Medicines Classifications Committee sought clarification on how nicotine in vaping products was regulated under the Medicines Act (1981). The MoH Vaping Regulatory Authority (VRA) responded via letter and stated, ‘Our position on vaping products is that their purpose is principally recreational rather than therapeutic and that their use as a cessation aid for smokers is secondary’.^39^ This statement contrasted with previous statements by the MoH that the primary role of e-cigarettes was as tools to support smokers to switch to less harmful alternatives.

Later that year, the industry sent the then Minister of Health a letter to oppose the VRA’s interpretation of set nicotine levels,^40^ which the VRA was trying to enforce. The industry argued that the VRA interpretation of the law was incorrect and that nicotine salt levels were too low. The letter was signed by leading vape brands and the manager of a tobacco company. The letter demonstrates active collaboration between tobacco and vape companies.

### 2023: regulations proposed to curb youth vaping and industry litigation

In response to continued increases in vaping among youth, the Labour Government introduced regulations which included a restriction on flavour names, requirements for child safety mechanisms, removable batteries and reduced nicotine levels in 2023. The government described these changes as an ‘effective ban on disposable vapes’.^41^ Proximity restrictions were also introduced to prevent new specialist vape retailers from opening within 300m of schools, sports grounds, and other specific community locations. Owners of several e-cigarette brands opposed the proposed nicotine reduction and applied to the high court to overturn the decision to reduce the permitted nicotine levels in August 2023.^42^ The judge dismissed the application later that year, and the nicotine limits and other regulations were reduced.

As a result of the 2023 general election, a new coalition government was formed. One of the first actions brought by the newly elected coalition was to repeal tobacco legislation brought in by the previous government, which stipulated an increase in the purchase age, reduced nicotine content and reduced retail supply of smoked tobacco products.

### 2024: lack of enforcement, tax reduction and new legislation

Despite specific e-cigarette legislation being in place for 4 years and high rates of use among people under the legal age of purchase of 18 years, the first vape retailer prosecution was not brought by the MoH until April 2024. The charge was brought for repeated offences by one retailer, including sales to minors and selling prohibited vape products.^43^ Charges were then dropped as the retailer left the country. Other retailers who have violated the law have also faced prosecution later that year.^44^

In 2024, the National Coalition Government reduced the excise tax on heated tobacco products by 50%^45^ against the advice of the MoH^46^ and WHO recommendations that Heated Tobacco Products (HTPs) be taxed at the same rate as combustible cigarettes.^47^

In September, the Coalition Government introduced a bill aimed at ‘preventing youth vaping’.^48^ The Smokefree Environments and Regulated Products Amendment Bill (No 2) proposed four key measures: a ban on disposable e-cigarettes, increased penalties for sales to minors, restrictions on the visibility of products from the street front and online, and including early childhood centres in the proximity restrictions (no new vape stores within 300 m).^48^ All measures will come into force in June 2025.

## Discussion

The findings of this research show that subsequent New Zealand Governments were slow to implement e-cigarette policies and that policies were often ambiguous and insufficient in addressing high rates of use among youth. Further, policy actions may have been influenced or driven by the industry’s vested interests, which played a significant role in opposing health-promoting policies, agenda setting and influencing social norms.

The WHO issued policy recommendations for e-cigarettes in 2014, outlining that if jurisdictions permit e-cigarettes, they should be governed by comprehensive policies to protect young people and nonsmokers.^26^ These recommendations included a suite of regulatory options, including, for example, restrictions on purchase age, advertising, sponsorship, product and use in public spaces. Importantly, the WHO stipulated that the tobacco industry and its allies must not be considered legitimate public health partners or stakeholders in e-cigarette legislation.^26^ In response to WHO recommendations, many jurisdictions began to introduce legislation. For example, the European Parliament issued a directive the same year,^9^ and nations, including the UK, had specific e-cigarette policies enacted by 2016.^49^ At the time, the New Zealand government considered e-cigarettes to be covered by existing legislation,^19,20^ but the MoH admitted that they were confusing and not routinely enforced.^22^ E-cigarette-specific legislation was not introduced until 2020, 6 years after the WHO directive, by which time daily use among 15- to 24-year-olds was 12.5%.

Our study highlights that when e-cigarette legislation was ultimately introduced in 2020, policies were inadequate to reduce youth vaping, as evidenced by increased prevalence and two further amendments to e-cigarette policies in 2023 and 2024. By this time, daily vaping among youth was over 20%.^11^ Due to insufficient policies, e-cigarette companies regularly circumvented restrictions to maximise profits, including store modifications,^50^ brand stretching^51^ and product diversification.^52^ One example of a weak policy is the policy on nicotine comparatively high permitted nicotine concentration, which, in 2020, was set to 50 mg/ml, more than double the limits set in countries such as the UK and Canada (20 mg/ml). Delayed and weak policies and a lack of enforcement^43,44^ may have substantially impacted social norms and uptake among New Zealand youth.

The research has also shown multiple interactions between the e-cigarette industry and public officials, which may have influenced policy. The MoH’s Electronic Cigarette Technical Expert Advisory Group, which included several members from the vape industry, facilitated engagement between industry representatives, officials and health advocates. This collaboration potentially influenced perceptions of e-cigarettes in smoking harm reduction and established the industry as a legitimate partner in reducing smoking. A later analysis found that three health organisations with members on the advisory group opposed multiple proposed e-cigarette policies, unlike most other New Zealand health organisations, who largely supported or called for more restrictive policies.^37^ Research also identified two e-cigarette retailers in the group actively marketed products using youth-oriented strategies such as music festival sponsorship and social media influencers^27^ while presenting their products as harm reduction tools to policymakers in submissions.^36^

Further interactions with the industry are also evident in this analysis. For example, the instance where a tobacco industry-funded organisation approached health sector actors to promote e-cigarette use for smoking harm reduction. The interactions, which prompted a sector-wide letter from the then Director General of Health, highlight the possible confusion within the health sector around the role of companies promoting tobacco harm reduction and a lack of understanding of WHO FCTC obligations.

Of significance is the unsuccessful 2018 court case brought by the MoH against a tobacco company. The tobacco industry has a history of overturning and opposing public health measures through litigation using significant financial resources.^53,54^ We argue that the period of more than 2 years, where no policies were in place, was crucial in normalising e-cigarette use in New Zealand through a combination of aggressive marketing and wide availability. The government-led promotional campaigns to promote vaping for smoking cessation may have also contributed to this normalisation, minimising the dangers of vaping and encouraging use among young people in New Zealand.^55^

Although subsequent New Zealand governments have played roles in the introduction and enforcement of delayed and weak policies, the newly elected Coalition Government, which came to power in 2023, has drawn heavy criticism for industry-friendly policy decisions, including the repeal of world-leading tobacco control policies aimed at equitably reducing smoking rates, reducing the taxes applied to heated tobacco products and proposing the legalisation of oral nicotine pouches.^56^ A tobacco company document from 2017 reveals that part of the tobacco company’s strategy to gain favourable regulation for its heated tobacco products was to lobby the political party NZ First, of which the associate minister of health is a member.^57^

## Limitations

Publicly available data limit this research/review and may not cover all relevant details, leading to potential gaps in the timeline. The more subtle influence of industry and covert lobbying cannot be quantified, as no regulations exist for lobbying in New Zealand.^58^

## Conclusion

This study examined and described New Zealand’s e-cigarette policies between 2015 and 2024. We focused on the occurrence of policy-relevant events, such as the introduction of regulations and legislation. We also documented instances of potential industry influence in the process between 2015 and 2024.

Our analysis revealed that New Zealand was slow to introduce e-cigarette-specific policies compared to other countries. When these policies were enacted, they were inadequate to curb the rising rates of vaping among youth. We identified key junctures where the tobacco and vape industry influenced, opposed and circumvented health-promoting policies using a harm reduction narrative. This narrative emphasised benefits to smokers who might use e-cigarettes to quit while simultaneously marketing to young people.

Furthermore, the MoH and subsequent New Zealand governments adopted harm reduction approaches and delivered ambiguous messaging around the purpose, safety and efficacy of e-cigarettes. Taken together, these findings highlight critical moments that may have contributed to the high prevalence of e-cigarette use among New Zealand youth. The findings underscore the need for policymakers to implement responsive and comprehensive policies on e-cigarettes and other emerging nicotine products. They also reiterate the importance of protecting public policies from industry and vested interests.

## Supplemental Material

sj-docx-1-rsh-10.1177_17579139251322009 – Supplemental material for An analysis of e-cigarette policy action, inaction and industry influence: implications for youth uptake in New Zealand Supplemental material, sj-docx-1-rsh-10.1177_17579139251322009 for An analysis of e-cigarette policy action, inaction and industry influence: implications for youth uptake in New Zealand by L Hardie, B Freeman and J McCool in Perspectives in Public Health

## References

[1] Sreeramareddy CT Acharya K Manoharan A . Electronic cigarettes use and ‘dual use’ among the youth in 75 countries: estimates from Global Youth Tobacco Surveys (2014–2019). Sci Rep 2022;12:20967. 36470977 10.1038/s41598-022-25594-4 PMC9722706

[2] Advertising Standards Authority UK. Advertising standards – vape advertising. Available online at: https://www.asa.org.uk/static/514432d6-9d8a-4e90-8c147a0e21c6416f/advertising-standards-vape-advertising-17092024.pdf (2024, last accessed 27 September 2024).

[3] Centers for Disease Control. Vaping and quitting. Smoking and tobacco use. Available online at: https://www.cdc.gov/tobacco/e-cigarettes/quitting.html (2024, last accessed 30 January 2025).

[4] Global Center for Good Governance in Tobacco Control. E-cigarette ban & regulation: global status as of October 2023, 2023. Available online at: https://ggtc.world/knowledge/novel-emerging-tobacco-products-and-product-regulation/e-cigarette-ban-regulation-global-status-as-of-october-2023

[5] Republic of Palau. RPPL No. 11-27, An Act to Amend Chapter 17, Title 11 of the Palau National Code to prohibit the importation, distribution, selling, possession, and use of electronic cigarettes within the Republic of Palau, and for other related purposes. Available online at: https://assets.tobaccocontrollaws.org/uploads/legislation/Palau/Palau-RPPL-No.-11-27-native.pdf (2023, last accessed 14 March 2024).

[6] Royal Government of Cambodia. National Authority for Combating Drugs. No. 001/14 Sor.Nor.No.NACD. (Unofficial Translation). Available online at: https://assets.tobaccocontrollaws.org/uploads/legislation/Cambodia/Cambodia-E-cig-Shisha-Circular.pdf (2014, last accessed 4 March 2024).

[7] Therapeutic Goods Administration. Australian Government. Changes to the regulation of vapes. Available online at: https://www.tga.gov.au/products/unapproved-therapeutic-goods/vaping-hub/changes-regulation-vapes (2023, last accessed 15 November 2024).

[8] Government of Japan. The Pharmaceutical Affairs Law. Japan. Available online at: https://www8.cao.go.jp/kisei-kaikaku/oto/otodb/english/houseido/hou/lh_02070.html (2020, last accessed 22 November 2023).

[9] European Parliament, Council of the European Union. Directive 2014/40/EU of the European Parliament and of the Council of 3 April 2014 on the approximation of the laws, regulations and administrative provisions of the Member States concerning the manufacture, presentation and sale of tobacco and related products and repealing Directive 2001/37/ECText with EEA relevance, 2014. 27660856

[10] Hammond D Reid JL . Smoking and vaping among youth and young adults: findings from the ITC youth & young adults surveys. Waterloo, Canada: University of Waterloo, 2024.

[11] Ministry of Health. New Zealand Health Survey 2023/24. Vaping at least once a day. Available online at: https://minhealthnz.shinyapps.io/nz-health-survey-2023-24-annual-data-explorer/_w_2ce21787/#!/explore-indicators (2024, last accessed 30 January 2025).

[12] Ministry of Health. New Zealand Health Survey – vaping/e-cigarettes – used at least one a day. Available online at: https://www.health.govt.nz/statistics-research/surveys/new-zealand-health-survey (2023, last accessed 26 February 2024).

[13] ASH. 2023 ASH year 10 snapshot topline smoking and vaping. Available online at: https://assets.nationbuilder.com/ashnz/pages/70/attachments/original/1702170472/2023_ASH_Y10_Snapshot_Topline_smoking_and_vaping_FINAL.pdf?1702170472 (2023, last accessed 23 January 2024).

[14] Ministry of Health NZ. Available online at: https://www.health.govt.nz/ (2024, last accessed 15 November 2024).

[15] New Zealand Parliament. New Zealand Parliament. Available online at: https://www.parliament.nz/en (2024, last accessed 15 November 2024).

[16] New Zealand Government. Beehive.govt.nz. Available online at: https://www.beehive.govt.nz/ (2024, last accessed 15 November 2024).

[17] Courts of New Zealand. Courts of New Zealand. Available online at: https://www.courtsofnz.govt.nz/ (2024, last accessed 15 November 2024).

[18] New Zealand Customs Service. Available online at: https://www.customs.govt.nz (2024, last accessed 15 November 2024).

[19] Smoke-free Environments Act. 108. Available online at: https://www.legislation.govt.nz/act/public/1990/0108/48.0/DLM223191.html (1990, last accessed 13 May 2022).

[20] Medicines Act. 1981.

[21] New Zealand District Court. Case 4478 Phillip Morris New Zealand Ltd v Ministry of Health.

[22] Ministry of Health. Policy options for the regulation of electronic cigarettes: a consultation document. Available online at: https://www.health.govt.nz/publication/policy-options-regulation-electronic-cigarettes-consultation-document (2016, last accessed 15 April 2020).

[23] Wagner N . Nicotine e-cigarettes to become legal. Available online at: https://www.beehive.govt.nz/release/nicotine-e-cigarettes-become-legal (2017, last accessed 17 March 2020).

[24] Ministry of Health. Technical Expert Advisory Group Terms of Reference, 2017. Available online at: https://www.health.govt.nz/system/files/2024-05/ecig-teag-tor-may2017.pdf

[25] Bonnett G . Third of vaping advisory group come from e-cigarette industry. RNZ, 3rd October 2019. Available online at: https://www.rnz.co.nz/news/national/400186/third-of-vaping-advisory-group-come-from-e-cigarette-industry (last accessed 24 March 2023).

[26] WHO Framework Convention of Tobacco Control – Conference of the Parties. WHO Framework Convention of Tobacco Control – Conference of the Parties Sixth Session: electronic nicotine delivery systems. Available online at: https://apps.who.int/gb/fctc/PDF/cop6/FCTC_COP6_10-en.pdf (2014, last accessed 7 May 2024).

[27] Hardie L McCool J Freeman B . E-cigarette retailers’ use of Instagram in New Zealand: a content analysis. Int J Environ Res Public Health 2023;20:1897. 36767263 10.3390/ijerph20031897 PMC9914635

[28] Hardie L McCool J Freeman B . Online retail promotion of e-cigarettes in New Zealand: a content analysis of e-cigarette retailers in a regulatory void. Health Promot J Austr 2022;33(1):91–8. 10.1002/hpja.464 33565666

[29] Health Promotion Agency. Vaping facts, 2019. Available online at: https://www.vapingfacts.health.nz/about-this-site.html

[30] Health Promotion Agency. QuitStrong. Te Hiringa Hauora/Health Promotion Agency. Available online at: https://www.smokefree.org.nz/quit/help-and-support/vaping-to-quit (2020, last accessed 29 October 2021).

[31] Espiner G . Big tobacco targeting Ma-ori with e-cigarettes. RNZ, 10 July 2019. Available online at: https://www.rnz.co.nz/news/in-depth/394073/big-tobacco-targeting-maori-with-e-cigarettes (last accessed 24 April 2022).

[32] Waa A Robson B Gifford H , et al. Foundation for a smoke-free world and healthy Indigenous futures: an oxymoron? Tob Control 2020;29:237–40. 10.1136/tobaccocontrol-2018-054792 PMC7042962 31076451

[33] Bloomfield A . Reminder about New Zealand’s international obligations regarding tobacco control and the need to avoid potential influence from tobacco companies, 2019. Available online at: https://www.health.govt.nz/strategies-initiatives/programmes-and-initiatives/smokefree-2025/who-framework-convention-on-tobacco-control/obligations-under-the-who-framework-convention-on-tobacco-control-fctc-article-53

[34] Health Committee. Final report of the Health Committee on Smokefree Environments and Regulated products (Vaping) Amendment Bill. Health Committe, New Zealand Government. Available online at: https://www.parliament.nz/resource/en-NZ/SCR_98007/3a8e520de6290062a2ea1b77545bc18f45bb68b7 (2 June 2020, last accessed 21 October 2020).

[35] New Zealand Parliament. Smokefree Environments and Regulated Products (Vaping) Amendment Bill. Available online at: https://www.parliament.nz/en/pb/bills-and-laws/bills-proposed-laws/document/BILL_94933/tab/submissionsandadvice (2020, last accessed 28 January 2021).

[36] Hardie L McCool J Freeman B . Use of supporting evidence by health and industry organisations in the consultation on e-cigarette regulations in New Zealand. PLoS ONE 2022;17:e0275053. 10.1371/journal.pone.0275053 PMC9522304 36174037

[37] Hardie L McCool J Freeman B . An analysis of key stakeholder policy perspectives in the proposed e-cigarette regulations in New Zealand. Health Promot J Austr 2023;34(4):875–82. 10.1002/hpja.709 36843364

[38] New Zealand Parliament. Smokefree Environments and Regulated Products (Vaping) Amendment Act 1990 (Amended 2020). 62. Available online at: http://www.legislation.govt.nz/act/public/2020/0062/latest/LMS313857.html (2020, last accessed 5 November 2020).

[39] Vaping Regulatory Authority, Ministry of Health. Response to the reclassification of nicotine in liquid preparations—67th meeting of the Medicines Classification Committee. Available online at: https://www.medsafe.govt.nz/profs/class/Agendas/Agen68/CommentsForAgenda68.pdf (2022, last accessed 9 March 2023).

[40] Multiple Vape Companies and British American Tobacco. Letter to the Hon. Dr Ayesha Verrall – interpretation of nicotine salt limits. Available online at: https://www.health.govt.nz/system/files/2024-05/2022-11-25_letter_to_the_hon_dr_ayesha_verrall_-_vra_interpretation_of_nicotine_salt_limits_in_vaping_products_additional_signatories_25.11.202_0.pdf (2022, last accessed 24 January 2024).

[41] LabourVoices. Release: more action to crack down on youth vaping. Available online at: https://www.labour.org.nz/news-action_crack_down_vaping_youth (2023, last accessed 26 September 2023).

[42] Ellis J . ALT NZ Ltd & Others v Attorney-General, 2023.

[43] Ministry prosecutes vape retailer| Ministry of Health NZ. Available online at: https://www.health.govt.nz/news/ministry-prosecutes-vape-retailer (2024, last accessed 15 November 2024).

[44] Ministry of Health. Series of prosecutions begins for illegal cigarette and vape sales. Available online at: https://www.health.govt.nz/news/series-of-prosecutions-begins-for-illegal-cigarette-and-vape-sales (2024, last accessed 23 September 2024).

[45] New Zealand Customs Service. Reduction in duty on heated tobacco products on 1 July 2024. Available online at: https://www.customs.govt.nz/about-us/news/important-notices/reduction-in-duty-on-heated-tobacco-products-on-1-july-2024/ (2024, last accessed 24 July 2024).

[46] Ministry of Health. Getting to Smokefree 2025: reform of vaping, smokeless tobacco and consumer nicotine product regulation. Available online at: https://www.health.govt.nz/system/files/documents/information-release/h2024034952_briefing_-_getting_to_smokefree_2025_reform_of_vaping_smokeless_tobacco_and_consumer_nicotine_product_regulation_black_box.pdf (2024, last accessed 24 July 2024).

[47] *Heated tobacco products: summary of research and evidence of health impacts* . Geneva: World Health Organization; 2023. Available online at: https://iris.who.int/bitstream/handle/10665/368022/9789240042490-eng.pdf?sequence=1 (last accessed 3 November 2023).

[48] Costello C . New Bill to crack down on youth vaping. Available online at: https://www.beehive.govt.nz/release/new-bill-crack-down-youth-vaping (2024, last accessed 15 November 2024).

[49] Government of the United Kingdom. The Tobacco and Related Products Regulations 2016. King’s Printer of Acts of Parliament. Available online at: https://www.legislation.gov.uk/uksi/2016/507/contents/made (2016, last accessed 7 May 2024).

[50] Boston A Robertson L Hoek J . Specialist vape store developments during the implementation of New Zealand’s Smokefree Environments and Regulated Products (Vaping) Amendment Act 2020. Tob Control 2023;32:e271–2. 10.1136/tobaccocontrol-2021-057123 35228319

[51] Cochran C Robertson L Hoek J . Online marketing activity following New Zealand’s vaping legislation. Tob Control 2023;32(2):263–4. 10.1136/tobaccocontrol-2021-056750 34301837

[52] Hardie L McCool J Freeman B . Industry response to New Zealand’s vaping regulations. Tob Control. Epub 2024 June 20. DOI: 10.1136/tc-2023-058427. PMC12128764 38429101

[53] Neuman M Bitton A Glantz S . Tobacco industry strategies for influencing European Community tobacco advertising legislation. Lancet Lond Engl 2002;359:1323–30. 10.1016/S0140-6736(02)08275-2 11965294

[54] Mandal S Gilmore AB Collin J , et al. Block, amend, delay: tobacco industry efforts to influence the European Union’s Tobacco Products Directive (2001/37/EC). Brussels: Smokefree Partnership/Cancer Research UK; 2009.

[55] Paewai P . ‘Our rangatahi deserve better’: experts urge vape shop restrictions to address ‘epidemic’. RNZ, 15 March 2023. Available online at: https://www.rnz.co.nz/news/te-manu-korihi/486006/our-rangatahi-deserve-better-experts-urge-vape-shop-restrictions-to-address-epidemic (last accessed 15 March 2023).

[56] Hoek J Ball J Waa A , et al. Mind the gap: Associate Health Minister’s actions conflict with Ministry advice, align with tobacco industry lobbying. Public Health Expert Briefing. Available online at: https://www.phcc.org.nz/briefing/mind-gap-associate-health-ministers-actions-conflict-ministry-advice-align-tobacco (2024, last accessed 16 November 2024).

[57] Leaked tobacco lobbying plan for ‘political pressure’ shows tobacco giant got its tax cut wish. RNZ, 23 August 2024. Available online at: https://www.rnz.co.nz/news/political/525810/leaked-tobacco-lobbying-plan-for-political-pressure-shows-tobacco-giant-got-its-tax-cut-wish (last accessed 16 November 2024).

[58] Chapple S Anderson T . Grease or sand in the wheels of democracy? The market for lobbying in New Zealand. Policy Q 2018;14:10–7.

