# Supplementary material for: An analysis of e-cigarette policy action, inaction and industry influence: implications for youth uptake in New Zealand
Source: Perspect Public Health. 2025 Mar 18;145(2):105–12. doi: 10.1177/17579139251322009 (PMC12069815; doi:10.1177/17579139251322009)
Supplement: sj-docx-1-rsh-10.1177_17579139251322009 – Supplemental material for An analysis of e-cigarette policy action, inaction and industry influence: implications for youth uptake in New Zealand [file sj-docx-1-rsh-10.1177_17579139251322009.docx]

# SUPPLEMENTARY TABLE

Supplementary Table 1 Timeline of the New Zealand regulatory process for e-cigarette legislation

| Date | Topic | Details |
| --- | --- | --- |
| 2015 | The New Zealand government regards e-cigarettes containing nicotine as prohibited from sale. | Existing legislation, established before the emergence of e-cigarettes ^1^, was regarded by officials to govern e-cigarette sales:   1. The Smoke-free Environments Act 1990 (SFEA) 2. The Medicines Act 1981.   The MoH considered the sale and supply of e-cigarettes containing nicotine prohibited if-   1. products contained nicotine (derived from tobacco) and were ‘oral tobacco products’ ^2^ or, 2. Medsafe had not approved the product for therapeutic use (i.e. smoking cessation). |
| 2016 | The MoH invites public submissions on potential regulation of e-cigarette products. | After reviewing evidence and concerns regarding e-cigarettes, the Government decided in principle to legalise the sale of e-cigarettes with appropriate controls as it concedes the current legislation is inadequate.  The MoH publishes a consultation document ^1^ outlining proposed options for regulating e-cigarette products and invites public feedback (2 August - 12 September 2016). Two hundred fifty submissions were received. |
| 2017, March 29^th^ | The government announces its intention to legalise e-cigarettes. | Associate Health Minister announces the Government’s intention to legalise and regulate the sale of e-cigarettes ^3^, applying restrictions to key areas;   1. Purchase age 2. Prohibit e-cigarette use where tobacco smoking is banned. 3. Advertising |
| 2017 | The MoH forms a technical advisory group. | The Electronic Cigarette Technical Expert Advisory Group was comprised of nine individuals who provided ‘technical expertise’ on e-cigarettes. Members include health experts and four industry actors. ^4^00/00/0000 00:00:00 |
| 2018, March 12^th^ | District court case MoH vs a tobacco company | Litigation was taken against a tobacco company by the Ministry of Health for the sale of the heated tobacco product in breach of the SFEA (1990) ^2^. The tobacco company challenges the charge, and the district court rules that SFEA (1990) does not extend to aerosol products ^5^.  A period of unrestricted sale and marketing of e-cigarettes commences as a result of this ruling. |
| 2018 October | MoH publishes a Regulatory Impact Statement | The MoH publishes the statement to inform decisions about proceeding with regulatory changes, outlining key objectives and proposed implementation of policies to improve the safety of e-cigarettes for smokers and protect young people from increased availability ^6^. |
| 2019 July | MoH implements Vaping Facts Website | The MoH, in partnership with the Health Promotion Agency and Smokefree Aotearoa 2025, implements the website *Vaping Facts* to promote the use of e-cigarettes as a safer alternative to combustible cigarettes ^7^ |
| 2019 August | The Director-General of Health cautions the health sector not to engage with the tobacco industry. | Tobacco industry affiliates approach District Health Boards to promote the use of heated tobacco products in smoking cessation. In response, the director general issued a letter directing the health sector not to engage with tobacco industry representatives^8^. |
| 2020 March | The NZ Parliament introduced The Smokefree Environments and Regulated Products (Vaping) Bill | NZ Parliament introduced a Bill to revise the Smoke-Free Environments Act 1990: the Smokefree Environments and Regulated Products (Vaping) Bill 2020 ^9^.  Key policies proposed include:   1. Minimum purchase age of 18 years 2. Extension of designated smokefree areas for e-cigarette use 3. Restrictions on advertising 4. Limit on flavours sold outside of ‘specialist’ retail settings. 5. Criteria set for specialist retailers (e.g., sales must be >80% e-cigarette products) |
| 2020 April | Public submissions on the Bill received | >1,000 written submissions received ^10^  84 Oral submissions ^11^  Key areas of response include advertising, purchase age, vapefree areas, packaging, and evidence. |
| 2020 June | MoH report to the Health Select Committee | The MoH summarised the public submissions and made recommendations to include the reduction of the threshold for ‘Specialist Vape Retailers’ to >70% of sales from e-cigarette products ^12^ |
| 2020 July | 2^nd^ reading of The Bill | The New Zealand House of Representatives debates the Bill. |
|  | Supplementary Order Papers introduced | Members propose amendments to the Bill, including   1. A reduction of the proportion of e-cigarette sales required for a retailer to be classified as a ‘specialist’ from >85% to >60%, 2. The prohibition of the use of e-cigarettes in vehicles carrying children in alignment with Prohibiting Smoking in Motor Vehicles Carrying Children Amendment Act 2020^13^ |
| 2020 August | 3^rd^ reading and Royal Assent | A summary of the Bill was presented to the House, and the formal process enacts the Bill into law, the Smokefree Environments and Regulated Products Act 1990 [SERPA, 1990]^14^ |
| 2020 November | Aspects of the SERPA commence | 1. The use of e-cigarettes is prohibited in workplaces, including in childhood education settings. 2. E-cigarette advertising and sponsorship are prohibited. The sale of e-cigarettes to persons under 18 years is prohibited. 3. Importation and sale of oral nicotine products is prohibited unless approved as a medicine. 4. Adverse reactions to e-cigarette products are required to be notified by the manufacturer to the Vaping Regulatory Authority 5. Retailers with total revenue of >50% from e-cigarette products can operate as ‘transitional specialist retailers’ ^15^ 6. Retailers who have not applied as a transitional specialist must operate as general retailers |
| 2021 January | MoH drafts vape regulations and seeks public consultation. | Two thousand five hundred twenty-one submissions were received ^16^.   1. Warning labels in Te Reo Maori are mandated. 2. Safety measures are defined. 3. Annual reporting is mandated. 4. Fees for product notification are reduced from $140 to $50 per year) 5. No additional amendments to the Act were applied to internal areas for e-cigarette use, or specialist vape retailer approvals. |
| 2021 May | Early childhood centres and colourings | Early childhood education centres must display ‘no smoking or vaping’ notices on-premises.  Colourings in e-cigarette products are prohibited ^17^ |
| 2021 August | SERPA takes effect | The Act is officially published in the gazette and highlights that general retailers are prohibited from selling e-cigarette flavours beyond tobacco, mint, and menthol that specialist and ‘transitional’ retailers are permitted to sell all flavours and manufacturers and importers are required to notify e-cigarette products. |
| 2021 | Retailer notices, display regulations and motor vehicle policies commence. The transitional period for retailers ends. | All e-cigarette retailers must display the minimum purchase age (R18) at the point of sale.  Approved product availability notices are required for display  Approved ‘harm reduction’ notices are permitted for display.  Manufacturer price lists must contain only permitted information.  E-cigarette use in cars carrying children is prohibited (to align with introduced legislation on smoking in vehicles)  Retailers who have not applied to be approved as specialist retailers revert to ‘general’ retailer provisions |
| 2022 May | Packaging regulations commence^18^ | Manufacturers must meet packaging and labelling regulations. |
| 2022 | Vaping Regulatory Authority position on e-cigarettes | The Vaping Regulatory Authority states that “Our position on vaping products is that their purpose is principally recreational rather than therapeutic and that their use as a cessation aid for smokers is secondary” ^19^. |
| 2023 January | Consultation measures to reduce the appeal of e-cigarettes to youth | As part of the Smokefree Environments and Regulated Products (Smoked Tobacco) Amendment Act, which came into force on 1 January 2023 ^20^. The Ministry of Health seeks public consultation on measures aimed at reducing youth vaping, including:   1. Restrict flavour names to reduce appeal, 2. Disposable e-cigarettes would be required to have a safety mechanism to prevent accidental use, removable or replaceable batteries, warning labels on the device, reduced nicotine levels from 50mg/mL to 35mg/mL and must be displayed in mg/mL rather than percentage. 3. Proximity restrictions on Specialist Vape Retailers include proximity to schools or sports grounds—no changes to general retail. |
| 2023 August | The government releases vape regulations to limit youth vaping. ^21^ | Regulations include:   1. All disposable vapes require a removable battery (for December 2023). Reusable vapes will require removable batteries by October 2024. 2. Flavour names will be restricted (March 2024) 3. Nicotine levels will be reduced to 20mg/ml for disposable vapes and 28.5mg/ml for reusable vapes (March 2024) 4. Child safety mechanisms are mandated. 5. Proximity restrictions on Specialist Vape Retailers include proximity to schools or sports grounds—no changes to general retail. |
| 2023 August | The vape industry initiates a High Court case against MoH to oppose nicotine reduction. | Applicants request to prevent the introduction of regulations reducing the maximum nicotine strength of reusable nicotine salt vaping products from 50 mg/mL to 28.5 mg/mL, pending the determination of their substantive application for judicial review ^23^. The judge dismissed the application for interim orders. |
| 2023 December | SERPA (Smoked Tobacco) Repealed | The National Coalition government repealed the Smokefree Environments and Regulated Products (Smoked Tobacco Act). ^24^ |
| 2024 April | First vape retailer prosecution is brought by the MoH | Vape retailer charged with repeated sales to minors and selling prohibited vape products. Charges are dropped as vendor left the country.^25,26^ |
| 2024 July | Taxes are reduced for heated tobacco products | National Coalition government reduces the excise tax on heated tobacco products by 50%.^27,28^ |
| 2024 October | Legislation is introduced to reduce youth access to vaping products | Smokefree Environments and Regulated Products Amendment Bill (No2)^29^ is introduced to ‘better protect children and young people by reducing their access to vaping products’ through   - A ban the manufacture, sale, supply, and distribution of disposable vapes - increased penalties for unlawful sale of regulated products to minors - imposing restrictions on the visibility of vaping products in retail stores and online advertising - including early childhood education centres in the proximity restrictions for specialist vape retailers.^30^ |

**References**

1. Ministry of Health. *Policy Options for the Regulation of Electronic Cigarettes: A consultation document*, https://www.health.govt.nz/publication/policy-options-regulation-electronic-cigarettes-consultation-document (2016, accessed 15 April 2020).

2. Smoke-free Environments Act. 108, https://www.legislation.govt.nz/act/public/1990/0108/48.0/DLM223191.html (1990, accessed 13 May 2022).

3. Wagner N. Nicotine e-cigarettes to become legal, https://www.beehive.govt.nz/release/nicotine-e-cigarettes-become-legal (2017, accessed 17 March 2020).

4. Ministry of Health. New Zealand’s history of vaping regulation.

5. New Zealand District Court. Case 4478 Phillip Morris New Zealand Ltd v Ministry of Health.

6. Ministry of Health. *Regulatory Impact Statement - Supporting smokers to switch to significantly less harmful alternatives*. New Zealand Government, https://www.health.govt.nz/system/files/documents/information-release/ris-support-smokers-to-switch-to-alternatives-jan-2019.pdf (2018, accessed 24 April 2022).

7. Health Promotion Agency. Vaping Facts, https://www.vapingfacts.health.nz/about-this-site.html (2019).

8. Bloomfield A. Reminder about New Zealand’s international obligations regarding tobacco control and the need to avoid potential influence from tobacco companies, https://www.health.govt.nz/system/files/documents/pages/dg-letter-to-ceos-re-tobacco-companies.pdf (2019).

9. New Zealand Parliament. Smokefree Environments and Regulated Products (Vaping) Amendment Bill, https://www.parliament.nz/en/pb/bills-and-laws/bills-proposed-laws/document/BILL_94933/tab/submissionsandadvice (2020, accessed 28 January 2021).

10. Hardie L, McCool J, Freeman B. Use of supporting evidence by health and industry organisations in the consultation on e-cigarette regulations in New Zealand. *PLOS ONE* 2022; 17: e0275053.

11. Ministry of Health. *Smokefree Environments and Regulated Products (Vaping) Amendment Bill Departmental Report*. Ministry of Health, https://www.parliament.nz/resource/en-NZ/52SCHE_ADV_94933_HE8844/87a2d74c9de673f4a8db2eabf3d8b1a8ca7ea7d4 (2020, accessed 22 March 2021).

12. Health Committee. *Report on the Smokefree Environments and Regulated Products (Vaping) Amendment Bill*. Wellington: Ministry of Health.

13. House of Representatives. Supplementary Order Paper Tuesday, 4 August 2020 Smokefree Environments and Regulated Products (Vaping) Amendment Bill.

14. Smokefree Environments and Regulated Products (Vaping) Amendment Bill 2020: Digest 2613 - New Zealand Parliament, https://www.parliament.nz/en/pb/bills-and-laws/bills-digests/document/52PLLaw26131/smokefree-environments-and-regulated-products-vaping (2020, accessed 15 November 2024).

15. About the Smokefree Environments and Regulated Products (Vaping) Amendment Act. *Ministry of Health NZ*, https://www.health.govt.nz/our-work/regulation-health-and-disability-system/regulation-vaping-and-smokeless-tobacco-products/about-smokefree-environments-and-regulated-products-vaping-amendment-act (2022, accessed 24 April 2022).

16. New Zealand, Ministry of Health. *Smokefree Environments and Regulated Products Act 1990 proposals for regulations: summary of submissions.*, https://www.health.govt.nz/system/files/documents/publications/submission_summary_report_for_publication.pdf (2021, accessed 31 May 2022).

17. New Zealand Parliament. Smokefree Environments and Regulated Products Act. 108, 2020.

18. Reddy P. Smokefree Environments and Regulated Products Regulations 2021.

19. Vaping Regulatory Authority, Ministry of Health. Response to the reclassification of nicotine in liquid preparations – 67th meeting of the Medicines Classification Committee, https://www.medsafe.govt.nz/profs/class/Agendas/Agen68/CommentsForAgenda68.pdf (2022, accessed 9 March 2023).

20. Proposals for the Smoked Tobacco Regulatory Regime – Public Consultation Document.

21. New youth vaping regulations set out | Beehive.govt.nz, https://www.beehive.govt.nz/release/new-youth-vaping-regulations-set-out (accessed 19 January 2024).

22. Waterman I, Marek L, Ahuriri-Driscoll A, et al. Investigating the spatial and temporal variation of vape retailer provision in New Zealand: A cross-sectional and nationwide study. *Soc Sci Med* 2024; 349: 116848.

23. Ellis J. ALT NZ Ltd & Others v Attorney-General. 2023.

24. Costello C. Smokefree Environments and Regulated Products Amendment Bill 22-1 (2024), Government Bill (Repeal of Smokefree Environments and Regulated Products (Smoked Tobacco) Amendment Act 2022), https://www.legislation.govt.nz/bill/government/2024/0022/latest/whole.html (2023, accessed 15 November 2024).

25. Vape shop owner flees NZ while on charges for selling to minors. *1News*, https://www.1news.co.nz/2024/04/17/vape-shop-owner-flees-nz-while-on-charges-for-selling-to-minors/ (accessed 13 May 2024).

26. Ministry prosecutes vape retailer | Ministry of Health NZ, https://www.health.govt.nz/news/ministry-prosecutes-vape-retailer (2024, accessed 15 November 2024).

27. Espiner G. NZ First Minister Casey Costello orders 50% cut to excise tax on heated tobacco products. *RNZ*, 18 July 2024, https://www.rnz.co.nz/news/in-depth/522429/nz-first-minister-casey-costello-orders-50-percent-cut-to-excise-tax-on-heated-tobacco-products (18 July 2024, accessed 18 July 2024).

28. New Zealand Customs Service. Reduction in duty on heated tobacco products on 1 July 2024, https://www.customs.govt.nz/about-us/news/important-notices/reduction-in-duty-on-heated-tobacco-products-on-1-july-2024/ (2024, accessed 24 July 2024).

29. Costello C. Smokefree Environments and Regulated Products Amendment Bill (No 2). 2024.

30. Costello C. New Bill to crack down on youth vaping, https://www.beehive.govt.nz/release/new-bill-crack-down-youth-vaping (2024, accessed 15 November 2024).
